# The Effects of Precursors on the Morphology and Chemical Mechanical Polishing Performance of Ceria-Based Abrasives

**DOI:** 10.3390/ma15217525

**Published:** 2022-10-27

**Authors:** Yuanyuan Zheng, Ning Wang, Zongyu Feng, Xianmin Tan, Zhenyu Zhang, Huiqing Han, Xiaowei Huang

**Affiliations:** 1 National Engineering Research Center for Rare Earth Materials, GRINM Group Co., Ltd., Beijing 100088, China; 2 GRIREM Advanced Materials Co., Ltd., Beijing 100088, China; 3 GRIREM Hi-Tech Co., Ltd., Langfang 065201, China; 4 Hebei Xiongan Rare Earth Functional Materials Innovation Center Co., Ltd., Baoding 071700, China

**Keywords:** ceria-based abrasives, rare earth precursors, chemical mechanical polishing, TFT-LCD glass substrates

## Abstract

Ceria-based abrasives are widely used in precision chemical mechanical polishing (CMP) fields, such as thin film transistor liquid crystal display (TFT-LCD) glass substrates and integrated circuits, because of their excellent physicochemical properties. Rare earth carbonates, as precursors of ceria-based abrasives, directly affect the morphology of ceria-based abrasives, which, in turn, affects the material removal rate (MRR) and the surface roughness (R_a_) after polishing. Herein, rare earth carbonates with different morphologies were obtained by adjusting reaction parameters during precipitation, including flake, spindle, and spheroid. Moreover, the phase of precursors was analyzed, and the evolution process of morphology from precursors to ceria-based abrasives was investigated. Furthermore, the effect of precursors on the polishing performance of ceria-based abrasives was explored. The results show that the primary particles of ceria-based abrasives are near-spherical, but the morphology and dispersion of the secondary particles are obviously inherited from precursors. Among them, near-spherical ceria-based abrasives prepared by nearly monodisperse near-spherical precursors show better uniformity and higher dispersion, and they not only achieve the lowest R_a_ but also obtain a higher MRR of 555 nm/min (9 wt.%) for polishing TFT-LCD glass substrates. The result is significant for the further optimization and application of high-performance ceria-based abrasives.

## 1. Introduction

Ceria-based abrasives are an important CMP-polishing material, which are widely used in polishing glass, the substrate of materials devices, and other silica-containing materials, due to its uniform particle size, moderate hardness, high polishing efficiency, and excellent polishing quality [1,2,3,4,5,6]. It has been paid attention to glass substrate polishing because of its greater application prospects. The TFT-LCD glass substrates are used in the thin film transistor liquid crystal display, which is a key strategic material in the electronic information display industry. Their surface machining accuracy is directly related to the display panel’s resolution, transmittance, and other key performance indicators [7,8]. The TFT-LCD glass substrates are divided by the area in the display industry, and it is generally considered that the 6th generation line and below is the low generation, and the 8.5 generation line (2200 mm×2500 mm) and above is the high generation. The high generation conforms to the development trend of the future large-screen and multi-screen era, and market demand is increasing. The high-generation TFT-LCD glass substrate is thin and soft, so the polishing accuracy is extremely strict. A single piece of high-generation glass substrate has an area of more than 5.5 m^2^ and a thickness of only a few hundred micrometers. The polished glass substrate is required to have high flatness and smoothness. Furthermore, the requirements for the performance of ceria-based abrasives are getting more stringent, including a higher MRR and an extremely low R_a_ [9]. Ceria-based abrasives and polished substrates polished exhibit chemical and mechanical interactions in the CMP process, and mechanical interactions play a dominant role [10,11,12]. The morphology of abrasives is a very important factor in the CMP process. It can significantly affect the polishing performance and CMP-induced defects, such as erosion and dishing [13]. Therefore, the morphology of ceria-based abrasives needs to be further optimized because of its significant impact on polishing performance.

Currently, the commonly used preparation method of ceria-based abrasives mainly includes three processes, which are precipitation, fluorination, and calcination [14,15,16]. However, high-precision classification is adopted to reasonably control the size of ceria-based abrasives [17,18]. The morphology and structure of ceria-based abrasives determine their polishing performance, so the influence of different process parameters on ceria-based abrasives has been studied extensively [19]. The fluorination can improve the dispersity of particles and the ceria-based abrasives’ MRR for the glass by introducing the fluridizer [20,21]. The fluorine (F) atoms can significantly improve the chemical activity of ceria in the polishing process, as well as enhance its polishing performance, and it can form LaOF and LaF_3_. However, LaF_3_ with low hardness is easily crushed during polishing, which lowers the MRR and shortens the useful life of ceria-based abrasives. Hydrofluoric acid (HF) is widely used as a fluridizer because of its better reaction rate and reaction uniformity, but excessive addition can damage the polished glass surface and increase the R_a_ [22]. In addition, calcination directly impacts phase structures and surface properties of ceria-based abrasives [19,23,24]. It was found for the ceria-based abrasives that the MRR increases with the calcination temperature. In the temperature range of ca. 300–700 °C, not only do the physical characteristics of ceria-based abrasives change dramatically, but so do their chemical activities [25,26]. However, there are still relatively few studies that demonstrate clear relationships of precursors, ceria-based abrasives, and polishing performance.

Rare earth carbonates are precipitation precursors, which are widely used in the industrial production of ceria-based abrasives. Previous research has shown that there are many types of rare earth carbonates, including rare-earth carbonates (RE_2_(CO_3_)_3_) [27], hydroxycarbonates (REOHCO_3_) [28], oxycarbonate hydrates (RE_2_O(CO_3_)_2_·H_2_O), and di-oxycarbonates (RE_2_O_2_CO_3_) (where RE represents a rare-earth ion) [29,30,31], which have been studied extensively at the nano- and micro-scale. In addition, rare earth carbonates with a broad range of morphologies can be obtained by different synthetic methods and experimental conditions, including spherical particles, triangular microplates, nanorods, nanoplates, etc. [32]. The urea homogenous precipitation method was the first technique employed to synthesize monodispersed spherical particles of rare earth carbonates [33]. Nanorods and nanoplates of rare earth carbonates can be obtained by altering counter anions [34]. Rare earth carbonates as precursors of ceria-based abrasives, their phase structures, and morphologies directly affect the physicochemical characteristics of abrasives and further affect their polishing rate and polishing precision. However, ordinary ceria-based abrasives are commonly prepared by flake-like rare earth carbonates in industrial production, which is the main reason for the low polishing accuracy.

Here, this work focus on the effects of rare earth carbonates on the morphology and chemical mechanical polishing performance of ceria-based abrasives. Rare earth carbonates with different phase structures and morphologies were synthesized by altering conditions during precipitation, where ammonium bicarbonate (NH_4_HCO_3_) and lanthanum-cerium sulfate mixed liquid ((Ce_0.7_, La_0.3_)_2_(SO_4_)_3_·xH_2_O) were used as raw materials, and La was added to improve the polishing performance of abrasives [35,36]. Then, ceria-based abrasives were obtained by fluorating and calcinating rare earth carbonates with different shapes, and the effect of precursors on the morphology of ceria-based abrasives was studied. Furthermore, the CMP process of ceria-based abrasives prepared by different precursors was evaluated, and TFT-LCD glass substrates were used as polishing workpieces. The effect of precursors on the polishing performance of ceria-based abrasives was investigated.

## 2. Materials and Methods

### 2.1. Chemicals and Materials

Ceria carbonate tetrahydrate (Ce_2_(CO_3_)_3_·4H_2_O, 99.9% purity) was purchased from Jiangsu Guosheng New Material Co., Ltd. (Taizhou, China). Lanthanum oxide (La_2_O_3_, 99.9% purity) was obtained from Shanghai Sinian Metal Material Co., Ltd. (Shanghai, China). Sulfuric acid (H_2_SO_4_, A.R.) was supplied by Beijing Chemical Works. NH_4_HCO_3_ (A.R.) was purchased from Tianjin Guangfu Technology Development Co., Ltd. (Tianjin, China). HF (Electronic grade, UP, 49 wt.%) was obtained from Jingrui Electronic Materials Co., Ltd. (Suzhou, China). All chemicals were used, as received, without further purification. Deionized water was purified by a water purification system (RO-DI plus, Hitech).

### 2.2. Synthesis of Ceria-Based Abrasives

**Figure 1:**
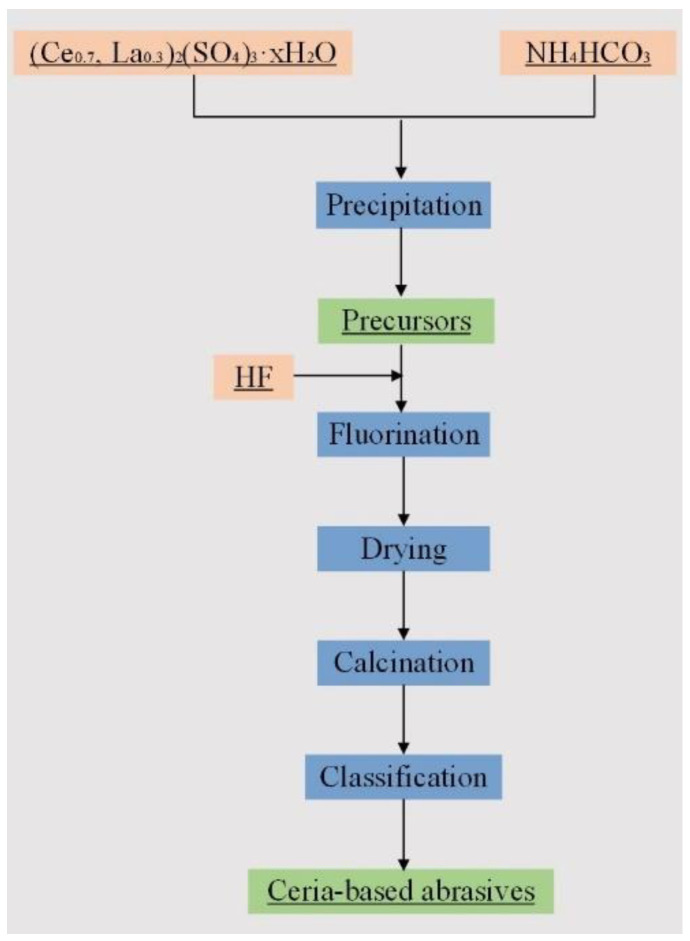
Schematic diagram of the synthesis of ceria-based abrasives.

In a typical preparation procedure, Ce_2_(SO_4_)_3_·xH_2_O was prepared by dissolving Ce_2_(CO_3_)_3_·4H_2_O in H_2_SO_4_. Similarly, La_2_(SO_4_)_3_·xH_2_O was formulated by dissolving La_2_O_3_ in H_2_SO_4_. (Ce_0.7_, La_0.3_)_2_(SO_4_)_3_·xH_2_O of 0.10 mol/L was obtained by mixing a certain amount of Ce_2_(SO_4_)_3_·xH_2_O with La_2_(SO_4_)_3_·xH_2_O, and the molar ratio of Ce to La is 2/1. Then, NH_4_HCO_3_ of 1.20 mol/L was employed as the precipitant, and (Ce_0.7_, La_0.3_)_2_(SO_4_)_3_·xH_2_O was used as the raw material. The total reaction molar ratio of precipitant to raw material was 3.1/1. Rare earth carbonate precursors were synthesized by parallel feeding in precipitation. In addition, precursors with different morphologies and phases were obtained in different precipitation temperatures and the aging process. Then, a certain amount of HF solution (0.5 mol/L) was added to the reaction system, and the molar ratio of F to La is 1.65. The fluorinated product was filtered and washed after aging for 2 h, and the filter cake was dried at 100 °C for 12 h. Finally, the fluorinated product after drying was calcinated at 900 °C for 12 h with a heating rate of 3 °C/min to obtain ceria-based abrasives. Furthermore, ceria-based abrasives with a narrow particle size distribution were obtained by classification. The schematic diagram of the synthesis steps is shown in Figure 1.

### 2.3. Polishing Measurements

The polishing process of ceria-based abrasives for TFT-LCD glass substrates was performed by a high-precision laboratory polishing machine (ProLap-15). Ceria-based abrasives were applied in the form of an abrasives slurry with different mass percentages. For each experiment, TFT-LCD glass substrates with a thickness of 0.5 mm were cut to 20 mm × 25 mm as polishing samples, and they adhered to a ceramic disc for polishing. The polishing test was performed with a pressure of 95 kPa and a time of 10 min. The velocity of the polishing pad was 45 rpm, and the flow velocity of the abrasives slurry was 50 mL/min. After polishing, polishing samples were removed from the ceramic disc by heating, rinsed with alcohol, and dried for further testing. The MRR of TFT-LCD glass substrates was determined via gravimetry from the mass loss during the polishing experiment, and the results were expressed in terms of the linear removal rate of TFT-LCD glass substrates in nm/min. In addition, scratches were observed and recorded on the surface of TFT-LCD glass substrates by a white light interferometer.

### 2.4. Characterization

The size and morphology of ceria-based abrasives were observed with the scanning electron microscope (SEM) (SEM, JEOL, Akishima, Japan, JSM-7900F). The phase analysis of ceria-based abrasives was achieved by an X-ray diffractometer (XRD, Smart-lab, Tokyo, Japan, 9KW) equipped with Cu-Kα radiation. The scanning range was 10–90°, and the rate was 4°·min^−1^. The particle size distribution of ceria-based abrasives was measured by a laser granularity analyzer (MS3000) after dispersing into the deionized water. The R_a_ and the polished surface state of the TFT-LCD glass substrates polished were measured by a white light interferometer (ZYGO NewView9000).

## 3. Results and Discussion

**Figure 2:**
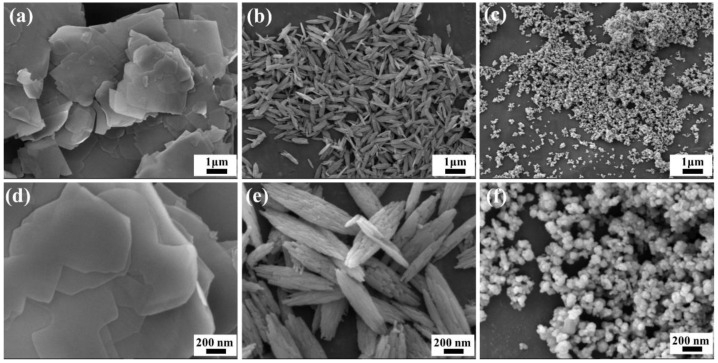
SEM images of precursors. (**a**,**d**): F-precursors; (**b**,**e**): S-precursors; (**c**,**f**): N-precursors.

Figure 2 shows SEM images of rare earth carbonates obtained by different precipitation conditions, which are flake-like precursors (F-precursors), spindle-like precursors (S-precursors), and near-spherical precursors (N-precursors). It can be seen that F-precursors are made up of many irregular thin sheets stacked (Figure 2a,d). S-precursors and N-precursors have uniform size and morphology, where the former has an average length of ~900 nm and a diameter of ~200 nm, and the latter has an average particle size of ~50nm. Moreover, N-precursors are nearly monodispersed. The synthesis of rare earth carbonates with different morphologies was achieved by controlling precipitation reaction conditions.

**Figure 3:**
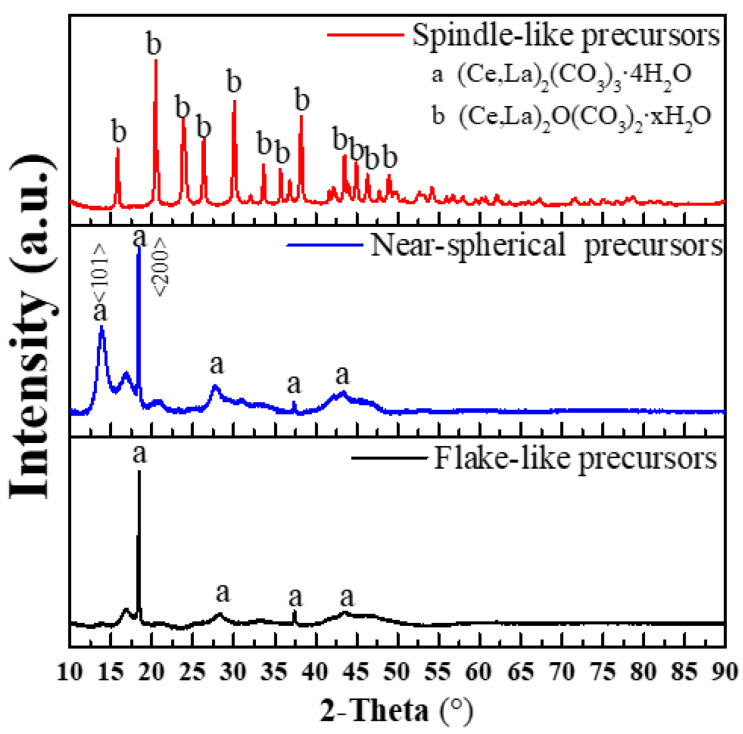
XRD diffraction patterns of different precursors.

XRD diffraction patterns of precipitation precursors in Figure 3 are shown to further analyze the phase structures. F-precursors and N-precursors are rare earth carbonate ((Ce, La)_2_(CO_3_)_3_·4H_2_O, JCPDS 00-006-0076), while S-precursors are mainly composed of rare earth oxycarbonate hydrate ((Ce, La)_2_O(CO_3_)_2_·xH_2_O, JCPDS 00-044-0617). Figure 3 shows the presence of amorphous phases for the N-precursors and F-precursors because of the low precipitation temperature, and it can be solved by calcination. Extensive literature indicates that different factors have effects on the phase and morphology of rare-earth carbonate particles. The reaction temperature is a parameter that allows easy control over the size and shape of rare earth carbonate particles [33]. In this work, the escape rate of carbon dioxide from rare earth carbonates was accelerated to a certain extent by increasing the reaction temperature and (Ce, La)_2_O(CO_3_)_2_·xH_2_O were formed [23]. The phase structures of precursors with different morphologies are different. XRD results show that F-precursors have the stronger <200> crystal plane and the un-conspicuous <101> crystal plane than N-precursors. According to crystal growth theory, the shape of the crystal depends on the relative growth rate of each crystal face. In this work, it shows that the morphology of precursors is affected by the relative growth rate of each crystal face [37,38], and the crystal plane of F-precursors and N-precursors with identical phases grow differently, so the morphology of precursors is different.

**Figure 4:**
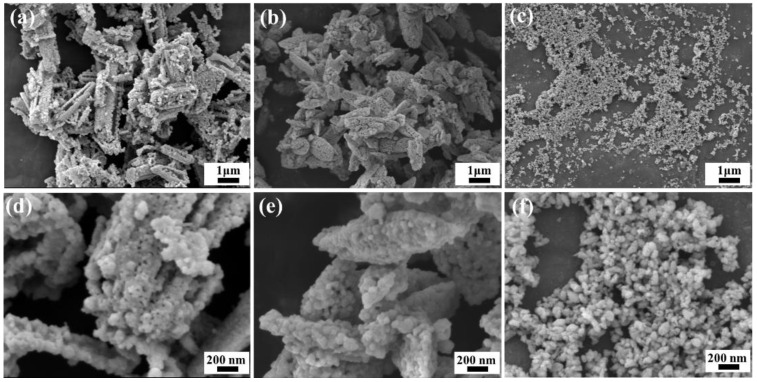
SEM images of ceria-based abrasives. (**a**,**d**): F-abrasives; (**b**,**e**): S-abrasives; (**c**,**f**): N-abrasives.

Furthermore, precursors with different morphologies and phases were fluorinated and calcined to obtain ceria-based abrasives. Figure 4 shows SEM images of ceria-based abrasives, which are flake-like (F-abrasives), spindle-like (S-abrasives), and near-spherical (N-abrasives), respectively. It can be seen from Figure 4 that the morphology and the dispersity of secondary particles about ceria-based abrasives are inherited from their precursors. However, the morphology of primary particles is almost near-spherical because of the etching of the HF solution. Moreover, primary particles’ sizes are close, and the average size is ~50 nm. This result may be attributed to the consistent conditions in the fluorination, such as the concentration and the additive amount of HF solution. Among them, the size of N-abrasives does not change obviously, compared with their precursors. The reason is that the particle size of near-spherical precursors is small (~50 nm) and close to the primary particle size of abrasives, and it is difficult to etch into smaller particles on the original basis during fluorination. In a word, rare earth carbonates affect the morphology of ceria-based abrasives, and there is an apparent inheritance relationship for secondary particles between them.

**Figure 5:**
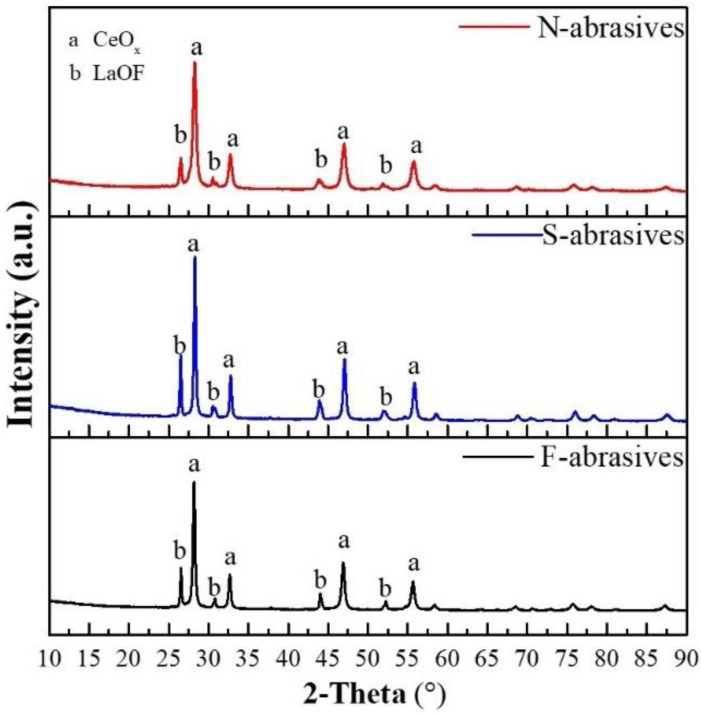
XRD diffraction patterns of ceria-based abrasives.

Figure 5 shows XRD diffraction patterns of ceria-based abrasives with different morphologies for which the diffraction peaks of CeO_x_ (JCPDS 00-004-0593) and LaOF (JCPDS 00-005-0470) could be indexed. Table 1 shows the phase information of abrasives, and the grain size of CeO_x_ obtained by the Scherrer formula. Although the intensity of CeO_x_ and LaOF of abrasives is different, the ratio of intensity of LaOF to CeO_x_ is close. The phases without low-hardness LaF_3_ are favorable to the CMP.

**Figure 6:**
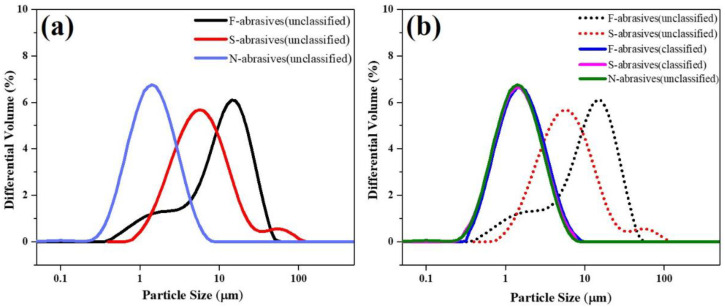
Particle size distributions of unclassified (**a**) and classified (**b**) ceria-based abrasives.

However, ceria-based abrasives tend to agglomerate after calcination, so further grading is a necessary process to achieve application requirements. Figure 6 shows particle size distributions of ceria-based abrasives with different morphologies before and after classification. As shown in Figure 6a, unclassified F-abrasives and N-abrasives have the largest and smallest size, respectively. Among them, the size distribution of S-abrasives with double peaks corresponds to their morphology. F-abrasives and S-abrasives have large particles after calcination, so they need to be further classified before the CMP. In this study, airflow classification is used to classify calcined products. The overall particle size distribution of graded F-abrasives and S-abrasives particles was consistent with that of ungraded N-abrasives particles (Figure 6b). The three kinds of abrasives have the same particle size distribution by adjusting airflow classification parameters. Further, the abrasives slurry with different concentrations was prepared. The polishing performance of ceria-based abrasives for TFT-LCD glass substrates was further evaluated.

**Figure 7:**
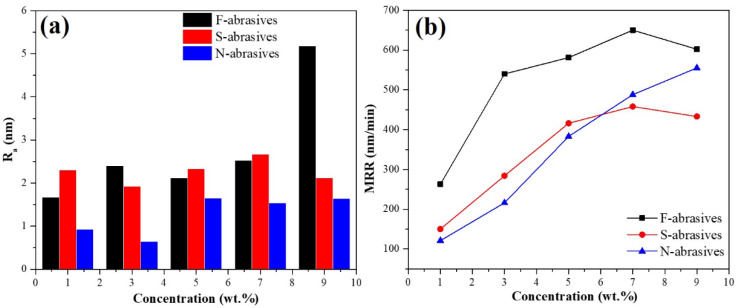
The R_a_ (**a**) and the MRR (**b**) of TFT-LCD glass substrates polished by the ceria-based abrasives slurry with different concentrations.

**Figure 8:**
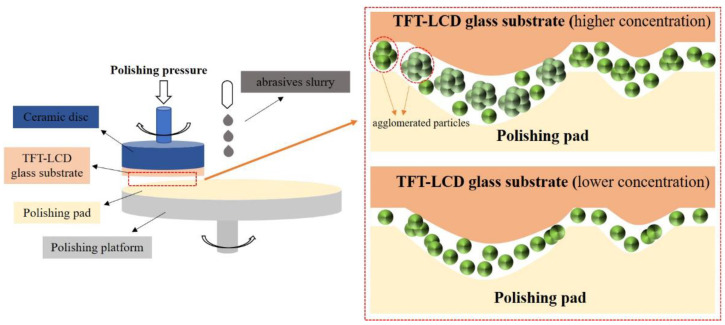
Schematic diagrams of the CMP process by using ceria-based abrasives in different concentrations.

Figure 7a shows the R_a_ of TFT-LCD glass substrates polished by the ceria-based abrasives slurry with different concentrations. In this study, the average height difference within a certain area (0.86 mm × 0.86 mm) was calculated as the R_a_. It can be seen from Figure 7a that N-abrasives always achieve a lower R_a_ than those of F-abrasives and S-abrasives in the concentration range of 1–9 wt.%. The R_a_ of F-abrasives and S-abrasives are more than 1.5 nm, but the R_a_ of N-abrasives is less than 1 nm at the appropriate concentration. Therefore, N-abrasives’ polishing quality is better because of their higher dispersion and homogeneity. Figure 7b shows the MRR of ceria-based abrasives with different morphologies. It can be seen that the MRR of three kinds of ceria-based abrasives increases with the increase in the slurry concentration. The MRR of F-abrasives is higher, and the MRR of N-abrasives is lower at the same concentration than F-abrasives. The shape of F-abrasives is irregular and angular, so the MRR is higher. By contrast, the morphology and size of N-abrasives are more uniform, so the MRR of N-abrasives is lower than F-abrasives at the same concentration. Their MRR increases with the increase in the concentration over a certain range, and the value of MRR reaches to 555 nm/min when the concentration is 9 wt.%. However, the MRR of F-abrasives and S-abrasives decreases with the increase in concentration from 7 wt.% to 9 wt.%. The main reason is that a higher concentration is easy to cause particle accumulation on the glass surface under experimental conditions, reducing the effective contact area [39,40]. Additionally, when the abrasives slurry concentration is high, the particle size after agglomeration is larger. Because the polishing pad is in direct contact with the raised parts of the TFT-LCD glass substrates, not all particles play a role (Figure 8). In addition, the polishing pad will produce recesses, and a number of small particles will be trapped under polishing pressure. The larger the functioning particles, the deeper the recesses will happen, and the small particles will be trapped and make no sense [41,42]. On the contrary, the proportion of functioned particles to all the particles in the slurry will increase due to the better uniformity and dispersity of abrasives slurry with lower concentration. Thus, high concentrations do not consistently increase the MRR for F-abrasives and S-abrasives, and the MRR of N-abrasives increases with the increase in the slurry concentration.

**Figure 9:**
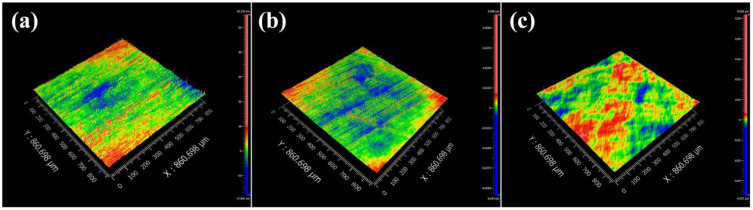
Interferometer images of TFT-LCD glass substrates polished by ceria-based abrasives. ((**a**) F-abrasives; (**b**) S-abrasives; (**c**) N-abrasives).

As an example, the experiment of polishing was taken by using abrasives slurry with a concentration of 7 wt.%, Figure 9 shows interferometer images of TFT-LCD glass substrates polished by ceria-based abrasives. Interferometer images present different height differences through color contrast, and the highest value and the lowest value can be obtained by using the corresponding software. As shown in Figure 9, glass substrates polished by F-abrasives and S-abrasives have a rougher surface, but fewer scratches appear on the surface of TFT-LCD glass substrates polished by N-abrasives. Because the secondary particles of F-abrasives and S-abrasives are flake-like and spindle-like, although their size distribution is close to N-abrasives after classification and primary particles are near-spherical. The edges of the particles are angular, which is easy to cause more scratches. The essence of the CMP process is the efficient coordination of chemical corrosion and mechanical wear, which is determined by the physical and chemical properties of abrasives as the main component of the polishing slurry. In this work, the size distribution of abrasives is closed after classification, and polishing parameters are the same. Experimental results are mainly related to mechanical wear, which is determined by the morphology of ceria-based abrasives. The morphology of ceria-based abrasives can significantly affect the MRR and the R_a_ [43]. Further, F-abrasive and S-abrasive with a morphology of edges and corners can obtain the higher MRR because of the greater force, but they also cause the presence of CMP-induced defects, for example, micro-scratches. Therefore, ceria-based abrasives with different morphologies exhibit different polishing performances. N-abrasives have a more uniform morphology, narrow size, and better dispersion, so they can achieve a higher MRR and lower R_a_. The polishing performance of N-abrasives is excellent.

## 4. Conclusions

Precipitation precursors with different morphologies and phases were obtained by altering the reaction conditions in the precipitation. The effect of precursors on the morphology of ceria-based abrasives was explored. The result shows that the morphology and dispersity of secondary particles inherit their precursors, and primary particles of abrasives are almost near-spherical with a size of ~50 nm because of the etching of HF solution. Therefore, rare earth carbonates with high dispersion and homogeneity are more conducive to obtaining high uniformity ceria-based abrasives.

Furthermore, the effect of precursors on the CMP performance of ceria-based abrasives was explored. High-performance ceria-based abrasives need to achieve a high MRR and good polishing quality (a low R_a_) at the same time. Although F-abrasives have a higher MRR (˃500 nm/min), more scratches (R_a_ ˃ 2 nm) are caused by their irregular morphology. By contrast, near-spherical ceria-based abrasives are beneficial to improving the polishing contact area and reducing mechanical damage. N-abrasives obtained by N-precursors have the characteristics of uniform morphology, narrow size, and high dispersion, which not only achieves a high MRR (9 wt.%, 555 nm/min) but also reaches excellent polishing quality to TFT-LCD glass substrates, including the lower R_a_ (˂1.5 nm) and fewer scratches. Therefore, rare earth carbonates with high dispersion and homogeneity are more conducive to obtaining high-performance ceria-based abrasives. N-abrasives have great application potential for the precision polishing of TFT-LCD glass substrates.

## Figures and Tables

**Table 1:** XRD phase information about LaOF and CeO_X_ of ceria-based abrasives.

Abrasives	Intensity-CeO_X_	Grain Size(nm)-CeO_X_	Intensity-LaOF	LaOF/CeO_X_
F-abrasives	23,153	43.6	4055	0.175
S-abrasives	30,392	51.5	5294	0.174
N-abrasives	25,687	44.0	4600	0.179

Note: LaOF/CeO_X_ is the ratio of intensity about their first strong peak.

## References

[1] Lim D.S., Ahn J.W., Park H.S., Shin J.H. (2005). The effect of CeO_2_ abrasive size on dishing and step height reduction of silicon oxide film in STI-CMP. Surf. Coat. Technol..

[2] Singh R.K., Bajaj R., Editors G. (2002). Advances in chemical-mechanical planarization. MRS Bull..

[3] Zantye P.B., Kumar A., Sikder A.K. (2004). Chemical mechanical planarization for microelectronics applications. Mater. Sci. Eng. R Rep..

[4] Krishnan M., Nalaskowski J.W., Cook L.M. (2010). Chemical mechanical planarization: Slurry chemistry, materials, and mechanisms. Chem. Rev..

[5] Pathak D., Bedi R.K., Kaur D. (2010). Characterization of AgInSe_2_ films deposited by hot-wall vacuum evaporation method. Mater. Manuf. Processes.

[6] Kim S., Bark C.W. (2020). Effect of Surface treatment by chemical-mechanical polishing for transparent electrode of perovskite solar cells. Energies.

[7] Ji M., Xin Y., Bo J. (2011). Structure and application of polarizer film for thin-film-transistor liquid crystal displays. Displays.

[8] Shiou A.C. (2003). Some technical aspects of glass substrates for TFT-LCD applications. Glass Technol..

[9] Lee H., Kim H., Jeong H. (2022). Approaches to sustainability in chemical mechanical polishing (CMP): A review. Int. J. Pr. Eng. Man-Gt..

[10] Cook L.M. (1990). Chemical processes in glass polishing. J. Non. Cryst. Solids.

[11] Sabia R., Stevens H.J. (2000). Performance characterization of cerium oxide abrasives for chemical-mechanical polishing of glass. Mach. Sci. Technol..

[12] Hoshino T., Kurata Y., Terasaki Y., Susa K. (2001). Mechanism of polishing of SiO_2_ films by CeO_2_ particles. J. Non-Cryst. Solids.

[13] Wu X., Lei H., Chen R.L. (2011). Preparation of porous alumina abrasives with different morphologies and their chemical mechanical polishing behavior. Adv. Mater..

[14] Chandrasekaran N. (2004). Material removal mechanisms of oxide and nitride CMP with celia and silica-based slurries—Analysis of slurry particles pre-and post-dielectric CMP. MRS Online Proc. Libr. (OPL).

[15] Liu H.J., Feng Z.Y., Huang X.W., Long Z.Q., Wang M., Xiao Y.F., Hou Y.K. (2013). Study on purification and application of novel precipitant for ceria-based polishing powder. J. Rare Earths.

[16] Huang S.D., Liu L.S., Li X.S., Li P.Z., Guo D.D. (2002). A Study on the preparation of rare earth polishing powder. Rare Earths.

[17] Wang X.L., Yi S.Z., Liang E.W., Wu Y.Y., Fang Z.X. (2013). Study on preparation of polishing powder for LCD. Adv. Mat. Res..

[18] Stachowiak G.P., Podsiadlo P., Stachowiak G.W. (2006). Shape and texture features in the automated classification of adhesive and abrasive wear particles. Tribol. Let..

[19] Kim E., Lee J., Park Y., Shin C., Kim T. (2020). Shape classification of fumed silica abrasive and its effects on chemical mechanical polishing. Powder Technol..

[20] Janoš P., Ederer J., Pilařová V., Henych J., Tolasz J., Milde D., Opletal T. (2016). Chemical mechanical glass polishing with cerium oxide: Effect of selected physico-chemical characteristics on polishing efficiency. Wear.

[21] Wang J., Jin Y.Z., Wang Z.B. (2019). Effect of fluoride incorporation on properties of rare earth polishing powders. Inorg. Chem. Ind..

[22] Yang G.S., Cui L.X., Xie B., Wu W.Y. (2016). Study on the effect of fluoride on the properties of ceria-based rare earth polishing powders. Rare Earth.

[23] Liu S.G., Zhang P., Li M., Hu Y.H., Wang M.T., Tao B. (2014). Study on fluorine behavior in pure cerium rare earth polishing powder. Rare Earth.

[24] Li X., Yang G., Wu W., Tu G. (2007). Study on roast reaction kinetics and crystal behavior of ceria-based rare earth polishing powder. J. Rare Earths.

[25] Jano P., Petrák M. (1991). Preparation of ceria-based polishing powders from carbonates. J. Mater. Sci..

[26] Li Y.X., Wang X.L., Ding L.M., Li Y., He R.H., Li J. (2022). Changing the calcination temperature to tune the microstructure and polishing properties of ceria octahedrons. RSC Adv..

[27] Pei W.L., Dong Z., Xiang H.C., Wang X.Y., Yang X.B., Wang J.J., Li Z.G., Zhou L.H. (2019). Evolution of the phases and the polishing performance of ceria-based compounds synthesized by a facile calcination method. RSC Adv..

[28] Qi R.J., Zhu Y.J., Cheng G.F., Huang Y.H. (2005). Sonochemical synthesis of single-crystalline CeOHCO_3_ rods and their thermal conversion to CeO_2_ rods. Nanotechnology.

[29] Qian L.W., Wang X., Zheng H.G. (2011). Controlled synthesis of three-fold dendrites of Ce(OH)CO_3_ with multilayer caltrop and their thermal conversion to CeO_2_. Cryst. Growth Des..

[30] Oikawa M., Fujihara S. (2005). Crystal growth of Ce_2_O(CO_3_)_2_·H_2_O in aqueous solutions: Film formation and samarium doping. J. Solid State Chem..

[31] Mihalache V., Secu M., Grivel J.C. (2018). Defect states and room temperature ferromagnetism in cerium oxide nanopowders prepared by decomposition of Ce-propionate. Mater. Chem. Phys..

[32] Mayama Y., Koyabu K., Masui T., Tamura S., Imanaka N. (2006). Synthesis of new red emitting phosphors based on rare earth oxycarbonates. J. Alloys Compd..

[33] Kaczmarek A.M., Van H.K., Van D.R. (2015). Nano-and micro-sized rare-earth carbonates and their use as precursors and sacrificial templates for the synthesis of new innovative materials. Chem. Soc. Rev..

[34] Zhang Y., Han K., Cheng T., Fang Z. (2007). Synthesis, characterization, and photoluminescence property of LaCO_3_OH microspheres. Inorg. Chem..

[35] Meher S.K., Rao G.R. (2012). Tuning, via Counter Anions, the morphology and catalytic activity of CeO_2_ prepared under mild conditions. J. Colloid Interface Sci..

[36] Praveen B., Cho B.J., Park J.G., Ramanathan S. (2015). Effect of lanthanum doping in ceria abrasives on chemical mechanical polishing selectivity for shallow trench isolation. Mat. Sci. Semicon. Proc..

[37] Jie C., Shuo H., Yang L., Wang T.Q., Xie L.L., Lu X.C. (2020). RE (La, Nd and Yb) doped CeO_2_ abrasive particles for chemical mechanical polishing of dielectric materials: Experimental and computational analysis. Appl. Surf. Sci..

[38] Krasinski M.J., Prywer J. (2007). Growth morphology of sodium fluorosilicate crystals and its analysis in base of relative growth rates. J. Cryst. Growth.

[39] Gu F., Wang Z., Han D., Guo G., Guo H. (2007). Crystallization of rare earth carbonate nanostructures in the reverse micelle system. Cryst. Growth Des..

[40] Wang Y., Zhao Y., An W., Ni Z., Wang J. (2010). Modeling effects of abrasive particle size and concentration on material removal at molecular scale in chemical mechanical polishing. Appl. Surf. Sci..

[41] Suratwala T., Steele W., Feit M., Shen N., Dylla-Spears R., Wong L., Phil M., Richard D., Selim E. (2016). Mechanism and simulation of removal rate and surface roughness during optical polishing of glasses. J. Am. Ceram. Soc..

[42] Wang L., Zhang K., Song Z., Feng S. (2007). Ceria concentration effect on chemical mechanical polishing of optical glass. Appl. Surf. Sci..

[43] Kim S.K., Yoon P.W., Paik U.Y., Katoh T., Park T.G. (2004). Influence of physical characteristics of ceria particles on polishing rate of chemical mechanical planarization for shallow trench isolation. Jpn. J. Appl. Phys..

